# Pattern of inpatient care for depression: an analysis of 232,289 admissions

**DOI:** 10.1186/s12888-020-02781-z

**Published:** 2020-07-16

**Authors:** Gernot Fugger, Thomas Waldhör, Barbara Hinterbuchinger, Nathalie Pruckner, Daniel König, Andrea Gmeiner, Sandra Vyssoki, Benjamin Vyssoki, Matthäus Fellinger

**Affiliations:** 1 Clinical Division of General Psychiatry, Department of Psychiatry and Psychotherapy, Medical University of Vienna, Vienna, Austria; 2 Centre for Public Health, Department of Epidemiology, Medical University of Vienna, Vienna, Austria; 3 Clinical Division of Social Psychiatry, Department of Psychiatry and Psychotherapy, Medical University of Vienna, Währinger Gürtel 18-20, 1090 Wien, Austria; 4 St. Pölten University of Applied Sciences, Sankt Pölten, Austria

**Keywords:** Depression, Gender, Sex, Age, Hospitalization, Length of stay

## Abstract

**Background:**

The prevalence of major depressive disorder (MDD) in women is up to 50% higher as compared to men. However, little is known about discrepancies in health care utilization between depressed female and male patients. Consequently, the aim of the present study was to elucidate gender differences regarding the frequency of hospital admissions and the length of inpatient treatment for MDD across the lifespan.

**Methods:**

This nationwide, registry-based study analyzed all inpatient admissions in psychiatric hospitals due to recurrent/non-recurrent MDD episodes according to ICD-10 (moderate (F32/33.1), severe (F32/33.2), severe with psychotic features (F32/33.3)) in Austria across 14 years. We calculated weekly admission rates per 100,000 patients by directly age-standardized rates.

**Results:**

Across 232,289 admissions (63.2% female) the population based admission rates in MDD were significantly higher in women (*p* < 0.001). Female to male ratios across subgroups were 1.65 (F32/33.1), 1.58 (F32/33.2), 1.73 (F32/33.3), and peaked around 65 years (ratio ≥ 2 for all subgroups). Length of hospital stay for women was significantly longer in all depression subtypes (*p* < 0.001).

**Conclusions:**

Elevated rates of inpatient treatment in women cannot solely be explained by a higher MDD prevalence and are dependent on age and type of depressive episode. Irrespective of the type and severity of the mood episode, women exhibit longer hospitalisation times.

## Background

Major depressive disorder (MDD) affected over 216 million people worldwide and was identified as one of the leading causes for years lived with disability according to the World Health Organization in the year 2015 [1, 2]. As repeatedly demonstrated in various patient samples women are significantly more often diagnosed with MDD [3], and female gender is associated with up to 50% higher prevalence rates of the disorder [4–6]. Clinical differences between women and men exhibiting MDD include, among others, the age of onset that was found to be earlier in the female population. Furthermore, atypical depression as well as comorbid anxiety disorders are more common in women whereas comorbid alcohol- and substance abuse are more frequently found in male patients with MDD [6, 7]. MDD is among the mental disorders with the highest suicide-mortality rates [8]. Even though existing studies revealed a higher rate of suicidal thoughts [9] and suicide attempts [6] in women, a male preponderance concerning more serious and fatal suicide attempts is referred to as the gender paradox in suicidal behaviour [10]. Furthermore, female patients suffering from MDD seem to report depressive symptoms more frequently and seek professional help more often than affected men [9, 11]. A possible explanatory model is depression-related stigma, which is markedly more common in men who are embarrassed to enter treatment [12]. Overall, evidence regarding gender differences in health care use, including admission rates and length of stay (LOS), in MDD is scarce [13, 14] and limited because of small sample sizes [15], a focus on seasonal patterns of admissions [16] or diagnostic uncertainties to other illnesses like bipolar disorder [17]. From a perspective on provision of health care, it is of value to elucidate whether the well-studied gender gap in depression can straightforwardly be transferred to a difference in admission rates. Discrepancies would indicate a varying need for inpatient care between depressed women and men that might be of consequence for decisions who to admit or discharge for instance. The same is true for the duration of inpatient stays. Since inpatient care accounts for almost half of the total MDD-related costs [18], understanding hospitalization rates for MDD is of economic relevance as well. Consequently, the main objective of the present study was to elucidate sex and age differences in admission rates to psychiatric hospitals of patients with recurrent and non-recurrent depressive episodes across the lifespan in Austria within a period of 14-years. Furthermore we aimed to detect a possible gender difference in LOS.

## Methods

### Design

We conducted a nationwide registry-based study concerning inpatient treatment in Austria for the time period 2003 to 2016. Data was provided in anonymized form by Statistics Austria, the national statistics agency. Statistics Austria collects data annually from the Austrian health system and provides data access for scientific research.

### Sample

For the current investigation, we examined data of all patients admitted to an adult or child and adolescent psychiatric hospital department with a diagnosis of a depressive disorder (ICD-10 F32/F33) as primary reason for inpatient treatment. Age groups were given in 5-year intervals for 15 to < 75 years and one group ≥75 years.

The dataset contained the following variables: depressive disorder (sub-)diagnoses (ICD-10 F32.0, F32.1, F32.2, F32.3, F32.8, F32.9 and F33.0, F33.1, F33.2, F33.3, F33.8, F33.9), sex, week of discharge, length of hospital stay in days, medical speciality and type of treatment care (acute vs. rehabilitation) of the attending department. Depression sub-diagnoses were grouped into moderate depressive episodes (ICD-10 F32/33.1), severe depressive episodes (F32/33.2) and severe depressive episodes with psychotic features (F32/33.3). Patients admitted to non-psychiatric departments or rehabilitation clinics, patients diagnosed with unspecified depressive episodes (ICD-10 F32.8, F32.9, F33.8, F33.9) as well as cases under the age of 15 years and cases exceeding inpatient treatment of 1 year (*n* = 31, 77.4% female) were excluded from further analysis. The reasons for the exclusion are explained hereinafter. Rehabilitation clinics in Austria have a determined inpatient treatment of at least three to a maximum of 6 weeks for all patients, which would have distorted the results. Patients from non-psychiatric departments were excluded from the analysis, as diagnostic recognition of MDD among non-psychiatric specialists is known to be low and inaccurate [19]. Diagnoses in psychiatric hospital departments are made by specialists and therefore show satisfactory validity. The diagnoses of other depressive episodes (F32/33.8), and depressive episode, unspecified (F32/33.9), lack the important information about depression severity. In this way, we sought to achieve a homogenous study sample of depressed individuals who are representative of acute inpatient psychiatric care.

### Statistical methods

Data management and analysis was done in SAS version 9.4 (SAS Institute Inc., Cary, NC, USA). Directly age standardized rates and corresponding 99.9% confidence intervals were calculated based on the European standard population [20] and by means of SAS procedure “proc stdrate” in order to adjust for a possible shift in the age distribution, comparisons of standardized rates by sex were done by rate ratios. Comparison of mean length of stay between groups was done by Wilcoxon signed rank test and described by mean, 1st,2nd and 3rd quartile. The overall significance level was set to 5%. Significance level for individual *p*-values was set to 5%/3 in order to adjust for multiple testing because of 3 sub-diagnosis groups.

## Results

The database included 232,289 admissions of patients with depression, *n* = 113,347 for moderate depressive episodes (ICD-10 F32/33.1), *n* = 91,993 for severe depressive episodes (F32/33.2) and *n* = 26,949 for severe depressive episodes with psychotic features (F32/33.3). Of an initial 411,069 admissions, altogether 178,780 were excluded from analysis to enhance the methodological quality as stated above. The mean age at admission was 46.8 years (SD 15.7) in men and 49.5 years (SD 17.1) in women. In total, 36.8% (*n* = 85,544) of the admissions were men (moderate: 36.7%, severe: 37.4% and severely depressed with psychotic features 35.2%). From a longitudinal perspective, only the number of admissions for moderate depressive episodes showed an upward trend, while the rates for severe depressive episodes and depressive episodes with psychotic features remained stable over time. Across the 14-year observation period, male and female admission rates showed a slight approximation. Data is displayed in Table 1.

**Table 1:** Number and percentage of inpatient admissions for depressive disorder subgroups according to sex for the time periods 2003–2007, 2008–2012, 2013–2016 and the total study period

Depressive subgroup (ICD-10)	Sex	2003–07 n (%)	2008–12 n (%)	2013–16 n (%)	Total 2003–16 n (%)
**Moderate** (F32/33.1)	Male	10,241 (35.9)	15,987 (36.4)	15,414 (37.7)	**41,642 (36.7)**
	Female	18,317 (64.1)	27,904 (63.6)	25,484 (62.3)	**71,705 (63.3)**
**Severe** (F32/33.2)	Male	10,316 (36.4)	12,910 (37.3)	11,201 (38.5)	**34,427 (37.4)**
	Female	17,992 (63.6)	21,707 (62.7)	17,867 (61.5)	**57,566 (62.6)**
**Severe with psychotic features** (F32/33.3)	Male	3066 (32.0)	3417 (36.0)	2992 (38.0)	**9475 (35.2)**
	Female	6518 (68.0)	6085 (64.0)	4871 (62.0)	**17,474 (64.8)**

The overall population based admission rates were significantly higher in women regarding all depressive episodes (*p* < 0.001), with a female to male rate ratio for moderate depressive episodes of 1.65, for severe depressive episodes of 1.58 and for severe depressive episode with psychotic features of 1.73 (Table 2).

**Table 2:** Admission estimate rate per 100,000 population and rate ratios by sex with 99.9% CI and *p*-values for depressive subgroups

Depressive subgroup (ICD-10)	Sex	Estimate Rate/ 100,000	99.9% CI^**a**^	Rate Ratio	99.9% CI^**a**^	***p***-value
**Moderate** (F32/33.1)	Female	138.8	137.1–140.6	1.65	1.62–1.69	**<.001**
	Male	84.1	82.7–85.5	ref.		
**Severe** (F32/33.2)	Female	111.7	110.2–113.3	1.58	1.54–1.61	**<.001**
	Male	70.8	69.5–72.1	ref.		
**Severe with psychotic features** (F32/33.3)	Female	34.1	33.3–35.0	1.73	1.65–1.80	**<.001**
	Male	19.8	19.1–20.5	ref.		

^a^*CI* Confidence interval

**Fig. 1:**
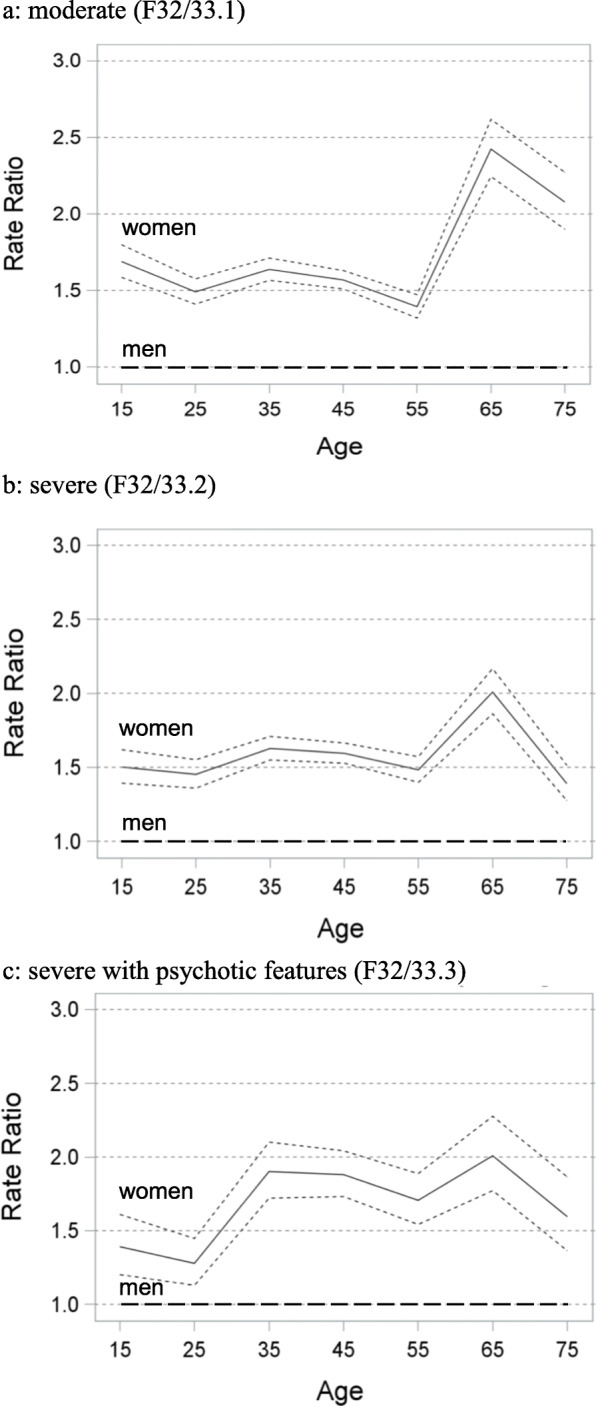
**a**-**c** Women’s rate ratio of inpatient episodes (continuous line) with 99.9%CI (broken lines) compared to men (rate: 1.0, dashed line) from age 15 to 75 in 10- year intervals for depressive subgroups according to ICD-10 (1a: moderate (F32/33.1), 1b: severe (F32/33.2), 1c: severe with psychotic features (F32/33.3))

Concerning the effect of age in patients with moderate and severe depressive episodes, a considerable increase in the female to male admission ratio was evident around the age of 55 years. Subsequently, the ratio peaked at the age of 65 years and gradually approached again thereafter. Population based admission rates for patients below the age of 55 appeared to be constant in these groups. Patients with MDD episodes with psychotic features showed a different pattern with a steady increase in the female to male admission rate until the age of 65 and a subsequent decrease thereafter. Figure 1 a-c display the population based women to men admission rates dependent on age. In addition, Suppl. Tab. 1, 2, 3 provide the total number of admissions and crude rates for women and men per age-group per 100,000 population.

The mean LOS for women in days (**d**) was significantly longer compared to men (18.6d, SD:18.5 vs. 17.4d, SD:17.7) during all depressive episodes (*p* < 0.001). Table 3 displays the average LOS for depression-subtypes separately, revealing significant gender differences across all groups.

**Table 3:** Comparison of the duration of inpatient treatment in days for male and female patients by depression severity

Depression subgroup (ICD-10)	Sex	Length of stay (d),				***P***-value
		Mean	SD^**a**^	95%CL^**b**^	Q1^**c**^/Q2^**d**^/Q3^**e**^	
**Moderate** (F32/33.1)	Male	14.83	14.49	14.70–14.97	5.0/12.0/20.0	**<.001**
	Female	15.80	14.86	15.70–15.91	6.0/13.0/22.0	
**Severe** (F32/33.2)	Male	19.04	19.32	18.83–19.24	6.0/15.0/26.0	**<.001**
	Female	20.33	19.97	20.16–20.49	7.0/16.0/28.0	
**Severe with psychotic features** (F32/33.3)	Male	22.44	22.37	21.98–22.89	8.0/17.0/29.0	**<.001**
	Female	24.06	24.31	23.70–24.42	9.0/19.0/31.0	

^a^*SD* Standard deviation

^b^*CL* Confidence level

^c^*Q1* Lower Quartile

^d^*Q2* Median

^e^*Q3* Upper Quartile

## Discussion

To the best of our knowledge, our study was the first to analyze a national sample of over 230,000 depressed individuals in order to elucidate differences regarding inpatient treatment patterns between women and men. Our results confirmed a female preponderance regarding hospital admissions due to depression. Overall, female to male admission rate ratios varied between 1.6 and 1.7 depending on the depression subtype. The admission rate ratio peaked around the age of 65 years. Furthermore, the duration of inpatient treatment was significantly longer in women.

The occurrence of depression is known to be higher in women than in men with a mean sex ratio of 2.1 for lifetime and 1.7 for point prevalence rates [5] which matches the female to male admission rate ratio exhibited in our study very well. Explanatory hypotheses for the gender gap in depression have been largely discussed. In fact, genetic influences seem to play only a minor role [21]. Hormonal fluctuations especially during puberty and menopause, alterations of the hypothalamic–pituitary–adrenal axis in terms of a blunted stress response as well as various psychosocial stressors like exposure to severe adversity like violence, discrimination and sexual abuse in women appear to be plausible concepts [22]. In addition, cultural and social differences between both sexes may contribute to the understanding of the gender-gap in depression. Firstly, there is substantial evidence that depression presents differently in male and female patients. Poor impulse control with anger attacks, for instance, appears to be guiding for male depression, whereas more typical symptoms including depressed mood, low appetite or sleep disturbance seem to be less common in men than in women [23]. The lack of recognition of a different symptomatology of masculine depression in the diagnostic criteria of ICD-10 could lead to an underdiagnosis of the disorder in men. Some authors deem the socialization of men according to a traditional gender role (men should avoid to show weakness for instance) to be responsible for a different presentation of depression and the gender disparity in help-seeking behaviour [24].

A recently published meta-analysis processing data of more than 1.7 million patients with depression revealed an overall odds ratio (OR) for gender difference in MDD of 1.95 representing a medium effect size. In fact, OR peaked already at the age of 13 to 15 years, decreased in the second decade of life and stayed stable thereafter [25]. Our study focusing on gender differences in admission and not prevalence rates revealed a slightly different picture. We found a peak regarding the female-to-male admission rate ratio around the age of 65 years before the ratio converged again until the age of 75 years. Even though our data do not provide information of individuals below 15 years a subtle decrease in the admission rate ratio is indicated at the age of 25 years analogous to the results of Salk and colleagues [25]. During the time span between 25 and 55 years the admission rate ratio remained relatively stable. A possible explanation for the increased admission rates in women compared to men around the age of 65 is delivered by a study describing increments of symptoms like motor agitation, feeling of guilt and hypochondriasis in women compared to men accompanied by enhanced levels of suffering [26]. The perimenopausal period as such might worsen depression by psychosocial challenges and menopause-related symptoms [27]. As a result women might seek inpatient treatment more frequently themselves and might be referred to hospital by their outpatient clinician more often due to an increased disease burden. A further hypothesis might lead towards different pathologies, namely Alzheimer’s disease (AD) and other dementia subtypes that tend to affect women to a larger extent than men [28]. A temporal relationship of AD and MDD has been discussed [29]. As far as sex differences in AD are concerned, Behavioral and Psychological Symptoms of Dementia including affective symptoms are disproportionally more often present in women [30]. At onset of AD, around the age of 65, less cognitive but affective symptoms might lead to hospital admissions in patients concerned, presumably under the guise of depression.

The gap in inpatient treatment episodes between women and men was most pronounced for psychotic depression compared to patients suffering from other depression subtypes. Even though a recent study found females to be affected by psychotic depression more often [31], large epidemiological studies were not able to detect significant differences in gender distribution [32]. The manifestation of severe psychotic symptoms in terms of hallucinations and delusions with disorganization, however, appear to be more prevalent in female patients [33]. A potentially higher symptom level and disease burden in women affected by MDD with psychotic features might be a reason for the more frequent inpatient treatment episodes in our data for that group.

Regarding the longitudinal aspect of our results, a trend towards a decreasing female to male rate ratio concerning admissions to hospital could be observed across the 14-year study period. This finding may be related to the hypothesis of a decreasing gender gap in MDD in populations that depart from traditional gender roles [34].

The duration of inpatient treatment was strongly affected by severity and subtype of depression ranging from a mean of 15 days for moderate depression to 24 days for depression with psychotic symptoms which is in line with literature of predictors of LOS [35]. Irrespective of the subtype of depression mean LOS was significantly longer in women than in men. Previous investigations with markedly smaller sample-sizes also found shorter inpatient stays in men but the outcome was not found to be better [16]. The hypothesis stating that women tend to suffer from more severe depression related to longer hospital LOS by nature [15] could be rejected in our sample since we detected a gender difference in LOS in moderately depressed patients as well. A systematic review investigating the impact of gender on hospitalization for depression found higher hospitalisation rates for women because they were significantly more likely than male patients to report their depression [36]. This is in line with a well documented gender difference in health seeking behaviour [37, 38]. Seen as a whole, in times of growing economic pressure and a limited number of hospital beds available, there is a danger that men are discharged too early because of a reluctance of reporting symptoms compared to women conceivably leading to early readmissions and a so-called revolving door effect [39].

When comparing our findings to inpatient treatment patterns for bipolar disorder (BD) in Austria it seems noticeable that women accounted for two thirds of all hospitalisations as well, despite an equally distributed life prevalence rate in BD. In contrast to unipolar depressed patients, LOS in bipolar depression was found to be shorter in women [40]. At first glance, these results appear counter-intuitive, a closer look points at the possibility that a large part of female admissions might be due to readmissions of a relatively small subgroup as described earlier in literature [39, 41].

A study based on service use data has several limitations including misclassification or over-selection of the severely ill. Since data were fully anonymized by Statistics Austria an identification of individuals within the data set was not possible. Consequently, further relevant information like the total number of inpatient stays per individual patient was unavailable. It is known that some factors leading to readmission for instance differ between female and male patients [42]. The influence of gender on the recurrence of depression is ambiguous in literature [7, 13]. In any case, missing information about the number of readmissions could potentially exert an effect on our findings regarding LOS. Consequently, it is not possible to fully rule out the fact that a shorter LOS in men is associated with more frequent readmissions in return. Inherent to the dataset providing information in five-year intervals, the cut-off point for inclusion was 15 years. Symptoms of MDD differ between adolescents and adults [43], which could impact the findings. However, the gender gap in prevalence of depression has consistently been detected from 14 years onwards [44]. Accordingly, we deem a cut-off age of 15 justifiable. The terms sex and gender were both used in conjunction with our findings, an approach we deem justifiable since differences found might be both biologically based (sex) and culturally based (gender). Even though this issue has been discussed for other psychiatric diseases before [45], existing literature does not always adhere to the proposed concepts and uses both terms under various aspects.

## Conclusions

For the first time national hospital discharge data was utilized to examine gender differences in hospitalisation patterns of depressed patients. In consideration of the known female preponderance regarding prevalence rates of depression, women appeared to be nonetheless overrepresented, especially within the age group of 65 years as well as in the subgroup of psychotic depression. Consequently, our findings are in line with previous research stating that women tend to utilize health services more frequently than men [46, 47]. Furthermore, mean LOS in women was significantly longer irrespective of the severity or subtype of the depressive episode. Whether the gender gap at age 65 is due to a diagnostic overlap of incipient neurodegenerative disorders or merely a result of a discrepant clinical presentation of depression in women and men is still in need of clarification. Certainly, clinicians should be aware of these considerations in the diagnostic process when treating patients of that age. In addition, the relative underrepresentation of depressed men in psychiatric inpatient services could be causally linked to the fact that male patients with MDD exhibit increased suicide rates [48]. Under this assumption, the indication for hospital admission in male patients with MDD should be even more closely examined. Anyway, a possible reluctance in utilizing inpatient care in men should not be encouraged by physicians in charge. In order to correctly identify assumed factors of causality, further research focusing on single cases are warranted.

## Supplementary information

**Additional file 1:Table S1.** Number of admissions and crude rates per 100,000 population for men and women per age and rate ratios by sex for F32/33.1

**Additional file 2:Table S2.**Number of admissions and crude rates per 100,000 population for men and women per age and rate ratios by sex for F32/33.2

**Additional file 3:Table S3.**Number of admissions and crude rates per 100,000 population for men and women per age and rate ratios by sex for F32/33.3

## Data Availability

The datasets used and/or analysed during the current study are available from the corresponding author on reasonable request. The authors had permission to use the anonymized database by the national statistics agency, Statistics Austria.

## Abbreviations

AD: Alzheimer’s Disease
BD: Bipolar Disorder
CI: Confidence Interval
d: Days
ICD-10: International Classification of Diseases, tenth edition
LOS: Length of Stay
MDD: Major Depressive Disorder
n: Number
SD: Standard Deviation
Suppl. Tab: Supplementary Table
Tab: Table
