# Supplementary material for: Pattern of inpatient care for depression: an analysis of 232,289 admissions
Source: BMC Psychiatry. 2020 Jul 16;20:375. doi: 10.1186/s12888-020-02781-z (PMC7364660; doi:10.1186/s12888-020-02781-z)
Supplement: Supplementary file 1 — Additional file 1:Table S1. Number of admissions and crude rates per 100,000 population for men and women per age and rate ratios by sex for F32/33.1 [file 12888_2020_2781_MOESM1_ESM.docx]

Suppl. Tab. 1: Number of admissions and crude rates per 100,000 population for men and women per age and rate ratios by sex for F32/33.1

| **Age** | **Men**  (n) | **Men**  (Crude Rate) | **Women**  (n) | **Women**  (Crude Rate) | **Rate Ratio** |
| --- | --- | --- | --- | --- | --- |
| 15 | 4866 | 67.1 | 7906 | 113.6 | 0.59 |
| 25 | 6434 | 82.3 | 9482 | 122.5 | 0.67 |
| 35 | 9534 | 104.2 | 15295 | 169.0 | 0.62 |
| 45 | 13010 | 147.9 | 20056 | 229.0 | 0.65 |
| 55 | 6551 | 98.1 | 9621 | 136.6 | 0.72 |
| 65 | 2674 | 53.3 | 7505 | 128.2 | 0.42 |
| 75 | 1892 | 56.8 | 7055 | 116.6 | 0.49 |
