# Supplementary material for: Pattern of inpatient care for depression: an analysis of 232,289 admissions
Source: BMC Psychiatry. 2020 Jul 16;20:375. doi: 10.1186/s12888-020-02781-z (PMC7364660; doi:10.1186/s12888-020-02781-z)
Supplement: Supplementary file 2 — Additional file 2:Table S2.Number of admissions and crude rates per 100,000 population for men and women per age and rate ratios by sex for F32/33.2 [file 12888_2020_2781_MOESM2_ESM.docx]

Suppl. Tab. 2: Number of admissions and crude rates per 100,000 population for men and women per age and rate ratios by sex for F32/33.2

| **Age** | **Men**  (n) | **Men**  (Crude Rate) | **Women**  (n) | **Women**  (Crude Rate) | **Rate Ratio** |
| --- | --- | --- | --- | --- | --- |
| 15 | 3237 | 44.6 | 4667 | 67.0 | 0.67 |
| 25 | 4184 | 53.5 | 6020 | 77.8 | 0.69 |
| 35 | 7344 | 80.3 | 11821 | 130.6 | 0.61 |
| 45 | 9688 | 110.1 | 15386 | 175.7 | 0.63 |
| 55 | 5238 | 78.4 | 8195 | 116.3 | 0.67 |
| 65 | 2679 | 53.4 | 6282 | 107.3 | 0.50 |
| 75 | 2057 | 61.8 | 5195 | 85.8 | 0.72 |
