# Supplementary material for: Pattern of inpatient care for depression: an analysis of 232,289 admissions
Source: BMC Psychiatry. 2020 Jul 16;20:375. doi: 10.1186/s12888-020-02781-z (PMC7364660; doi:10.1186/s12888-020-02781-z)
Supplement: Supplementary file 3 — Additional file 3:Table S3.Number of admissions and crude rates per 100,000 population for men and women per age and rate ratios by sex for F32/33.3 [file 12888_2020_2781_MOESM3_ESM.docx]

Suppl. Tab. 3: Number of admissions and crude rates per 100,000 population for men and women per age and rate ratios by sex for F32/33.3

| **Age** | **Men**  (n) | **Men**  (Crude Rate) | **Women**  (n) | **Women**  (Crude Rate) | **Rate Ratio** |
| --- | --- | --- | --- | --- | --- |
| 15 | 882 | 12.2 | 1178 | 16.9 | 0.72 |
| 25 | 1259 | 16.1 | 1594 | 20.6 | 0.78 |
| 35 | 1661 | 18.2 | 3124 | 34.5 | 0.53 |
| 45 | 2446 | 27.8 | 4581 | 52.3 | 0.53 |
| 55 | 1649 | 24.7 | 2968 | 42.1 | 0.59 |
| 65 | 983 | 19.6 | 2305 | 39.4 | 0.50 |
| 75 | 595 | 17.9 | 1724 | 28.5 | 0.63 |
